# *SIR2* Expression Noise Can Generate Heterogeneity in Viability but Does Not Affect Cell-to-Cell Epigenetic Silencing of Subtelomeric *URA3* in Yeast

**DOI:** 10.1534/g3.120.401589

**Published:** 2020-07-28

**Authors:** Jian Liu, Laureline Mosser, Catherine Botanch, Jean-Marie François, Jean-Pascal Capp

**Affiliations:** Toulouse Biotechnology Institute, University of Toulouse, INSA, CNRS, INRAE, Toulouse, France

**Keywords:** Epigenetics, *SIR2*, silencing, gene expression noise, viability

## Abstract

Chromatin structure clearly modulates gene expression noise, but the reverse influence has never been investigated, namely how the cell-to-cell expression heterogeneity of chromatin modifiers may generate variable rates of epigenetic modification. Sir2 is a well-characterized histone deacetylase of the Sirtuin family. It strongly influences chromatin silencing, especially at telomeres, subtelomeres and rDNA. This ability to influence epigenetic landscapes makes it a good model to study the largely unexplored interplay between gene expression noise and other epigenetic processes leading to phenotypic diversification. Here, we addressed this question by investigating whether noise in the expression of *SIR2* was associated with cell-to-cell heterogeneity in the frequency of epigenetic silencing at subtelomeres in *Saccharomyces cerevisiae*. Using cell sorting to isolate subpopulations with various expression levels, we found that heterogeneity in the cellular concentration of Sir2 does not lead to heterogeneity in the epigenetic silencing of subtelomeric *URA3* between these subpopulations. We also noticed that *SIR2* expression noise can generate cell-to-cell variability in viability, with lower levels being associated with better viability. This work shows that *SIR2* expression fluctuations are not sufficient to generate cell-to-cell heterogeneity in the epigenetic silencing of *URA3* at subtelomeres in *Saccharomyces cerevisiae* but can strongly affect cellular viability.

Noise in gene expression is an important contributor to phenotypic diversification and can have important consequences in processes ranging from the developmental to the evolutionary level (Ackermann 2015; Raj and van Oudenaarden 2008). However, its relationships with other processes that produce phenotypic diversity by acting at the genetic or epigenetic level are only partly understood. The influence of genetic and epigenetic variation on noise is well characterized (Sanchez *et al.* 2013; Sanchez and Golding 2013) but the reverse relationship has seldom been explored.

The level of expression noise is particularly influenced by promoter architecture. Genes with TATA-box containing promoters show higher noise levels than do other genes in yeast and mammals (Newman *et al.* 2006; Zhang *et al.* 2009; Zoller *et al.* 2015). Mutating the TATA box clearly decreases promoter-mediated noise (Blake *et al.* 2006; Fraser *et al.* 2004; Hornung *et al.* 2012). The strength, number and position of transcription factor binding sites also affect noise: promoters with more TF binding sites have higher expression noise for instance (Sharon *et al.* 2014; To and Maheshri 2010). Finally, another factor that has remarkable effects on expression noise is the positioning of nucleosomes on promoters. A promoter with nucleosome binding sites has silenced or open alternative states. The transient opening and reclosing of these promoters lead to transcriptional bursting events of variable duration and frequency, generating cell-to-cell variability in gene expression (Sanchez and Golding 2013). Promoters with polynucleosome-disfavoring sequences thus have less noise because higher transcription burst frequencies lead to lower cell-to-cell heterogeneity (Sharon *et al.* 2014).

These data suggest that epigenetic changes play an important role. Moreover, results from single-cell nucleosome mapping on the same promoter revealed significant cell-to-cell variation in nucleosome positions during induction of the *PHO5* gene in yeast (Small *et al.* 2014). Nucleosome positioning complexity may thus contribute to the flexibility and heterogeneity of gene expression, independently of the promoter sequence. A qualitative model for nucleosome positioning in *Saccharomyces cerevisiae* has shown that nucleosome architecture is indicative of the amount of transcriptional noise in TATA-box-containing promoters (Zaugg and Luscombe 2012). Also, increased eviction or sliding of promoter nucleosomes makes the promoter more accessible to transcriptional machinery in the initiation of transcription and thus probably reduces expression noise during induction (Rawal *et al.* 2018). Other results highlight the role of the chromatin environment in the modulation of expression noise: for instance, changing the location of a gene changes its level of noise in *S. cerevisiae* (Becskei *et al.* 2005), *Candida albicans* (Anderson *et al.* 2014) and chicken cells (Viñuelas *et al.* 2013). Mean expression and noise are uncorrelated across genomic locations in mammalian cells and the chromatin environment clearly influences the level of noise. More repressed chromatin is associated with higher expression noise (Dey *et al.* 2015). Mutations in chromatin remodelers also affect this phenomenon in yeast (Fraser *et al.* 2004; Weinberger *et al.* 2012). Finally, heterochromatin formation has been suggested to be locus autonomous and to be established stochastically in *S. cerevisiae* (Xu *et al.* 2006). Similarly, triggered spreading of heterochromatin is stochastic, multimodal, and fluctuates dynamically over time in fission yeast (Greenstein *et al.* 2018; Obersriebnig *et al.* 2016). However, another study has found that fluctuations in heterochromatic silencing are not locus autonomous (Mano *et al.* 2013), revealing a lack of consensus on this point. In any case, the lack of stability correlates with high histone turnover, showing that cell-to-cell heterogeneity in the silencing status of precise loci is mainly controlled by the dynamics of the chromatin environment (Greenstein *et al.* 2018; Obersriebnig *et al.* 2016).

In contrast, much less is known about how gene expression noise influences the rates of genetic and epigenetic modifications. Gene expression noise directly influences genetic variability in *Escherichia coli* because response to DNA alkylation damage and the subsequent mutagenesis depend on the stochastic expression of Ada (Uphoff *et al.* 2016); and in *S. cerevisiae*, where cell-to-cell heterogeneity in the homologous recombination rate stems from noise in the expression of genes such as *RAD52* and *RAD27* (Liu *et al.* 2019). However, the influence of noise in the expression of epigenetic regulators on chromatin modification activity has never been explored.

Sir2 is a well-known histone modifier of the Sirtuin family, largely characterized in *S. cerevisiae* for its ability to perform epigenetic silencing at silent mating-type loci (HML/HMR), telomeres, subtelomeres, and rDNA through its NAD+ dependent histone deacetylase activity (Grunstein and Gasser 2013). This well-conserved protein from eubacteria and archaea to man associates with Sir3 and Sir4 in the SIR complex to perform silencing in *S. cerevisiae* by acting on the N-terminal tails of histones H3 and H4 (Grunstein and Gasser 2013; Newcomb and Bedalov 2009). Interestingly, it has been shown to influence the expression noise level of subtelomeric *TLO* genes in *C. albicans* by modulating the shift between silent and active chromatin states (Anderson *et al.* 2014). In *S. cerevisiae*, epigenetic silencing processes are commonly studied using telomere silencing assays on strains in which either *URA3* or *ADE2* is inserted in subtelomeres in a previous *Δ**ura3* or *Δ**ade2* background (Grunstein and Gasser 2013). Typically, these assays are used to identify inhibitors of chromatin modifying enzymes (Newcomb and Bedalov 2009), notably for anticancer drug discovery (Simon and Bedalov 2004). Other loci are commonly used to test silencing. For instance, cellular levels of Sir2 regulate the extent of gene silencing observed at the rDNA (Smith *et al.* 1998).

*SIR2* thus provides a good model to test the influence of expression noise on epigenetic variability. We chose to investigate the epigenetic silencing of a *URA3* cassette inserted in subtelomeres as a function of cellular levels of Sir2. We show that variable Sir2 expression does not generate heterogeneity in the epigenetic silencing of subtelomeric *URA3* between cells. Nevertheless, *SIR2* expression noise creates heterogeneity in viability, which decreases progressively with increases in Sir2 levels across the whole expression distribution. Thus, while *SIR2* expression fluctuations are not sufficient to produce cell-to-cell heterogeneity in the epigenetic silencing of subtelomeric *URA3*, they can generate cell-to-cell heterogeneity in viability in *S. cerevisiae*.

## Material And Methods

### Yeast constructs and growth conditions

All the primers used in this article are listed in Table S1. The strain UCC2210 (Matα; *ade2**Δ*::*hisG*; *his3**Δ200*; *leu2**Δ0*; *lys2**Δ0*; *met15**Δ0*; *trp1**Δ63*; *ura3**Δ0*; *adh4*::*URA3**-TEL (VII-L)*; *ppr1*::*HIS3*) was used to measure *URA3* silencing activity at subtelomeres (frequency of *URA3* expression). To create the *Δ**sir2* strain, a PCR fragment containing the *LYS2* gene and homologous to *SIR2* was amplified from the genomic DNA of the S288c strain with primers D1 and D2, and transformed into UCC2210. The construct was verified by PCR with primers V1 and V2. To create the C-terminal fusion strain *Sir2*-tdTomato, a PCR fragment containing *tdTomato-KanR* and homologies of the end of *SIR2* was amplified with primers C1 and C2 from the plasmid pfa6a-*tdTomato-KanR* (made in our laboratory), and transformed into UCC2210. The constructs were verified by PCR with primers V3 and V4. The N-terminal fusion of *SIR2* was created using a two-step strategy, taking advantage of the selection and anti-selection of the *LYS2* gene. First, a PCR fragment containing the *LYS2* gene and homologous to the beginning of *SIR2* was amplified from the genomic DNA of the S288c strain with primers N1 and N2, and transformed into the strain UC2210. The constructs were verified by PCR with primers V5 and V2. Second, a synthesized fragment (Eurofins, Figure S1) containing tdTomato and homologous to the beginning of *SIR2* was transformed to the previous strain to create the N-terminal fusion (selection on alpha-aminoadipate). The construct was verified by PCR with primers V5 and V4.

To integrate an additional copy of *SIR2* in UCC2210, the *LEU2* gene was first reintroduced into the genome after amplifying *LEU2* from the S288C genome using primers V6 and V7 and integrating it into UCC2210. A PCR fragment containing the *SIR2* gene was then amplified from the genomic DNA of the S288c strain with primers V8 and V9 respectively containing the SalI et NotI restriction sites. *SIR2* was then inserted in the yeast chromosomal integration vector pJRL2 (Addgene) previously modified in our laboratory to replace the selection cassette *his-**URA3**-kanR-his* by *kanMX4* only (Liu *et al.* 2015) and digested using SalI and NotI. After ligation, the resulting plasmid was linearized by AscI digestion (restriction site between the regions homologous to the *LEU2* locus) and transformed in UCC2210 using the lithium acetate method. Recombinants were selected on YPD + G418 agar plates and insertion was verified by PCR with primers V10 and V11. The strain was called UCC2210+*SIR2**in**LEU2*.

For creating the tdTomato-Tsl1 strain, the *tdTomato* sequence was amplified by PCR from the plasmid pfa6a-*tdTomato-KanR* with primers overlapping 50bp downstream and upstream the *TSL1* start codon (primers T1 and T2, respectively). *tdTomato* was integrated using a CRISPR-Cas9 strategy. A plasmid carrying the gene coding for the Cas9 enzyme, *LEU2* for auxotrophic selection (derived from pML107, Addgene) and containing an appropriate gRNA obtained with primers T3 and T4 and inserted in the plasmid thanks to the SapI cloning site, was transformed in the BY4741 strain (MATa; *his3**Δ1*; *leu2**Δ0*; *met15**Δ0*; *ura3**Δ0*) together with *tdTomato* amplified with primers T1 and T2. PAM cleavage site was determined with the CRISPR Direct webtool for gRNA sequence design (https://crispr.dbcls.jp/).

All the strains were grown in liquid YNB medium (20 g/L glucose (Sigma), 1.71 g/L yeast nitrogen base without amino acids or nitrogen (Euromedex) and 5 g/L ammonium sulfate (Sigma)) supplemented with dropout solution corresponding to the selection marker at 30° with vigorous shaking (200 rpm).

The YPD plates used for viability measurements contained 20 g/L glucose, 20 g/L agar (Euromedex), 10 g/L peptone (Euromedex) and 10 g/L yeast extract (Euromedex). The SCD-ura plates used for *URA3* expression measurements contained 20 g/L glucose, 20 g/L agar, 1.71 g/L yeast nitrogen base, 5 g/L ammonium sulfate, and 0.77 g/L CSM-URA^-^ (Euromedex).

### Western blotting

Western blotting analyses were performed using standard protocols. Total cellular protein (100µg) was separated by electrophoresis in a 10% SDS–PAGE gel and transferred to nitrocellulose membrane. Sir2 was detected using a goat polyclonal antibody (yN-19, Santa Cruz Biotechnology) followed by incubation with horseradish peroxidase (HRP)-conjugated anti-goat IgG (Abcam). Equal loading was verified and normalization was performed on two replicates using stain-free imaging technology and the Image Lab software (Bio-Rad).

### Fluorescence activated cell sorting

An overnight culture was diluted 20 times and grown to exponential phase (6 h) to measure the fluorescence profile of the UCC2210, Sir2-tdTomato, tdTomato-Sir2, BY4741, Rad27-tdTomato and Rad52-tdTomato strains by MACSQuant VYB with the MACSQuantify Software (Miltenyi Biotec). A total of 10^5^ cells was analyzed for each strain, and the fsc files were exported and analyzed using the software R (v3.2) with the Bioconductor packages (v3.0). A norm2Filter filter was applied on FSC-A/SSC-A to select homogeneous cells in terms of size, shape, and cellular complexity. tdTomato fluorescence (Channel Y2-A) was log-transformed. All the figures were prepared using the transformed data. Each measurement was repeated three times.

The cell sorting experiments were performed on a MoFlo Astrios EQ cell sorter and analyzed with the Summit v6.3 software (Beckman Coulter). Cells in stationary phase were diluted 100 times and grown at 30° with vigorous shaking (200 rpm) for 16 h prior to cell sorting (final OD ≈ 2). Cultures were spun down at 3000g for five minutes at 4°. Growth media were removed and the cells were re-suspended in ice cold PBS. The SmartSampler and CyClone tube holders were kept at 4° during cell sorting. Cell sorting was carried out with a 70 µm nozzle at an operating pressure of 60 psi. The sorting speed was kept at around 30 000 events per second. The purity mode was chosen for sorting along with a single-drop droplet envelope. Single cells of similar size and granularity were first selected based on the FSC-Area *vs.* SSC-Area (488 nm laser) plot and the FSC-Height *vs.* FSC-Area (488 nm laser) plot. Then, based on the tdTomato fluorescence histogram (560 nm laser, 614/20 filter), single cells were simultaneously sorted either into the 2% most and least fluorescent (Figure 2A), or into five subpopulations defined in terms of their fluorescence as follows: 0–20%, 20–40%, 40–60%, 60–80% and 80–100% (only for viability analysis) (Figure 4B).

To analyze the dynamics of recovery of the initial gene expression profile from the sorted extreme subpopulations, the UCC2210:tdTomato-Sir2 strain was grown overnight at 30° in YPD medium and diluted 10 times in the morning. After 3 h, 6.10^5^ cells from the bottom cells and the top 2% were sorted simultaneously with MoFlo Astrios EQ (Beckman Coulter). The unimodality of the sorted cells was verified and they were then grown at 30° in YPD medium. The dynamics of expression recovery was monitored with a MACSQuant VYB flow cytometer (Miltenyi Biotec) for 6 h or 24 h.

### Measurement of survival frequency on SCD-ura plates and of viability on YPD plates

To measure the frequency of survival on SCD-ura plates in whole populations, 100 µL of 100 times diluted UCC2210, tdTomato-*Sir2* and UCC2210+*SIR2**in**LEU2* cultures (exponential phase at OD 1.5); and 100 µL of 10000 times diluted *Δ**sir2* and *Sir2*-tdTomato cultures (exponential phase at OD 1.5) were spread on SCD-ura plates. At the same time, 20 µL of 10000 times diluted culture was spread on YPD plates for all the strains. The plates were kept in a 30° incubator for 3 days and the number of clones was counted. As survival is most likely due to epigenetic *URA3* activation, we estimated the frequency of *URA3* expression for UCC2210, *tdTomato-**Sir2* and UCC2210+*SIR2**in**LEU2* as follows:

$$f=\frac{n_{-ura}\times 20}{n_{YPD}\times 10000}$$ (1)

where f denotes the frequency of *URA3* expression, $n_{-ura}$ denotes the number of clones on the SCD-ura plates, and $n_{YPD}$ denotes the number of clones on the YPD plates.

The frequency of *URA3* expression for *Δ**sir2* and *Sir2*-tdTomato was calculated as follows:

$$f=\frac{n_{-ura}\times 20}{n_{YPD}\times 100}$$ (2)

To measure the frequency of *URA3* expression in the sorted subpopulations with different expression levels, 10000 cells from each subpopulation were sorted and spread on SCD-ura plates, and 150 cells were then sorted and spread on YPD plates. The plates were kept in a 30° incubator for 3 days and the number of clones was counted. The frequency of *URA3* expression was calculated as follows:

$$f=\frac{n_{-ura}\times 150}{n_{YPD}\times 10000}$$ (3)

The viability was calculated as follows:

$$v=\frac{n_{YPD}}{n_{plated}}$$ (4)

where v denotes the viability, $n_{YPD}$ denotes the number of clones on the YPD plates and $n_{plated}$ denotes the number of plated cells to estimate viability.

All the experiments were repeated at least three times and three technical replicates were done for each sample in each experiment (the number of clones given in each experiment for each sample is the mean of these three technical replicates).

### Statistical analysis

Results were mainly compared using two-tailed Student’s *t*-tests on the data of three independent replicates. Results are presented as mean ± SD and *p* values as * *P* < 0.05, ** *P* < 0.01 and *** *P* < 0.005. The Pearson correlation coefficient between expression and viability was calculated using Microsoft Excel.

### Data availability

Strains are available upon request. Figure S1 shows the sequence of the synthetized *tdTomato* gene with flanking homologies to *SIR2*. Figure S2 illustrates the growth of clones with subtelomeric *URA3* either on SCD–ura plates or on 5-FOA plates. Figure S3 and S4 describe the recovery of the initial expression profile after cell sorting for both low and high Sir2-expressing cells, after 6 h and 24 h respectively. Figure S5 shows the viability of subpopulations of the tdTomato-Tsl1-tagged strain expressing extreme tdTomato levels similar than in the tdTomato-Sir2-tagged strain. Table S1 lists the primers used in this study. Table S2 contains the raw data of the silencing and viability analyses. Supplemental material available at figshare: https://doi.org/10.25387/g3.12724955.

## Results

### SIR2 expression noise does not generate cell-to-cell heterogeneity in the epigenetic silencing frequency of subtelomeric URA3

**Figure 1:**
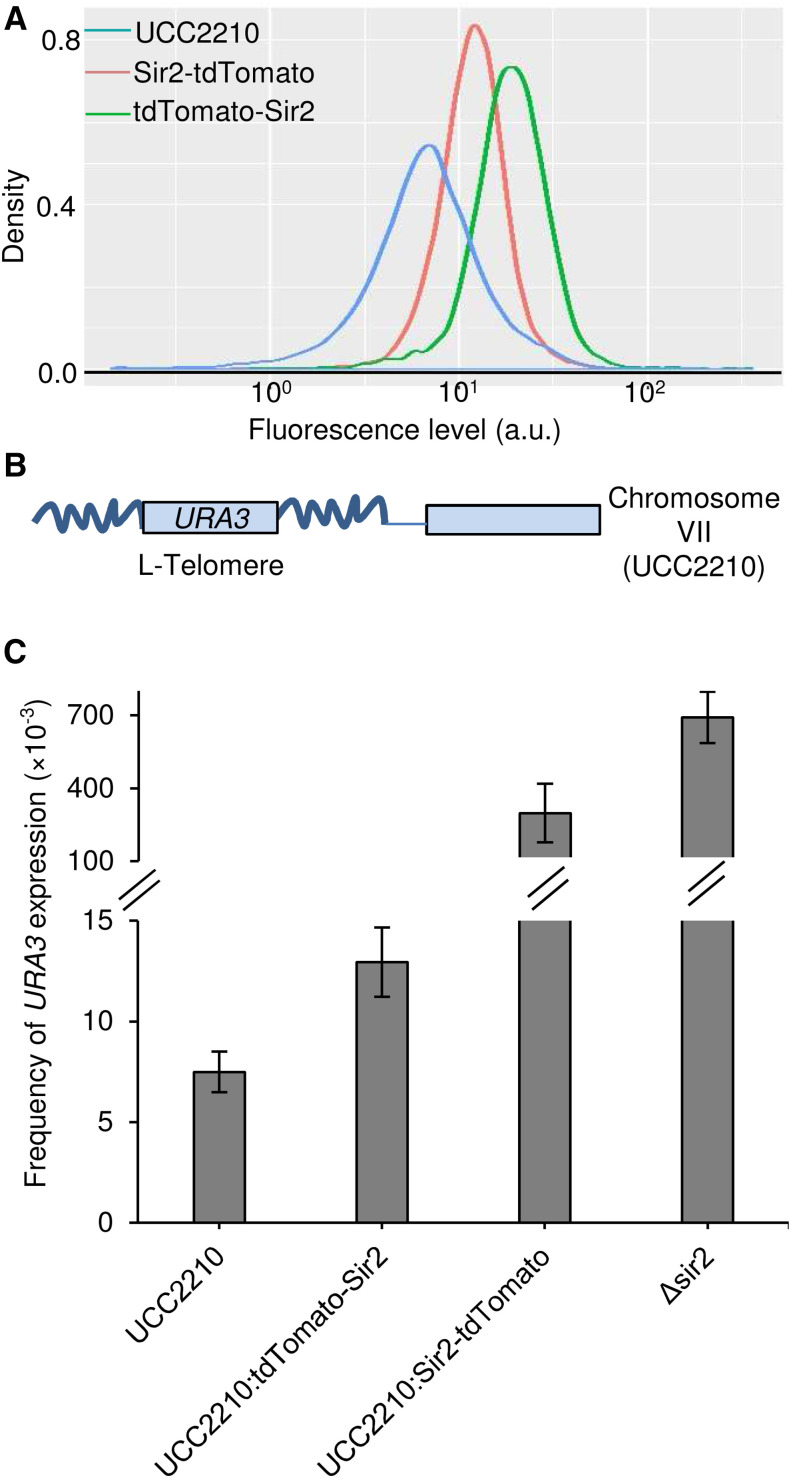
Fluorescence and subtelomeric *URA3* silencing frequency of strains expressing Sir2 fused to tdTomato., A) Fluorescence profiles of the non-fluorescent control UCC2210 containing the subtelomeric *URA3* silencing substrate, and of its derivatives with *SIR2* fused to tdTomato at its original genomic locus either N-terminally (tdTomato-*SIR2*) or C-terminally (*SIR2*-tdTomato). B) The subtelomeric *URA3* silencing substrate consists of a *URA3* gene inserted in the vicinity of a telomere where its transcription is modulated by epigenetic silencing. As the strain used lacks Ppr1, which is a strong activator of *URA3*, positive selection can be performed on uracil-deficient plates where only *URA3*-expressing cells can grow., C) Frequency of *URA3* expression for the non-fluorescent control UCC2210, N-terminal tagged tdTomato-Sir2 strain, C-terminal tagged Sir2-tdTomato strain, and *Δ**sir2* strain. The results shown are the means and standard deviations of three independent experiments.

As a fluorescent signal above the auto-fluorescence background was needed to efficiently sort Sir2-expressing cells, we chose tdTomato to tag Sir2 because it is one of the brightest fluorescent proteins available. We fused tdTomato either to the N-terminal or to the C-terminal domain of Sir2 to select the fused protein with the highest fluorescence and the best functionality. The fluorescence distribution of both fused proteins overlapped slightly with the auto-fluorescence background, and only slight differences in fluorescence were observed between the fusions, the N-terminal fusion being the most fluorescent (Figure 1A).

We also tested the functionality of the fused proteins in subtelomeric silencing assays by using the reporter strain containing *URA3* in the vicinity of a telomere (Figure 1B) where its transcription is modulated by epigenetic silencing (Grunstein and Gasser 2013). Silencing of *URA3* allows growth in the presence of 5-fluoroorotic acid (5-FOA), because the Ura3 gene product converts 5-FOA to 5-fluorouracil, an inhibitor of DNA synthesis and disruptor of RNA processing (Lum *et al.* 2004; Giaever *et al.* 2004) that causes cell death. The frequency of *URA3* repression can thus be scored accurately on plates that contain 5-FOA (Grunstein and Gasser 2013). On the contrary, if the strain lacks Ppr1, a strong activator of *URA3*, *URA3* is globally repressed and is only expressed at low levels. In the ppr1 strain, only perturbed silencing can permit sufficient *URA3* transcription for growth in a medium lacking uracil, but this occurs at low frequency and the whole population grows poorly in this medium (Newcomb and Bedalov 2009). This means that the frequency of *URA3* expression can be scored on plates that lack uracil (Grunstein and Gasser 2013).

The strain used in this work (UCC2210) lacks Ppr1. We therefore selected *URA3* expressing cells on uracil-deficient plates because only cells in which *URA3* is not silenced grow efficiently enough to form countable colonies on this medium (Figure S2). In our experimental conditions, the frequency of *URA3* expression in the UCC2210 strain with the *URA3* telomeric reporter was about 8.10^−3^, but increased markedly when *SIR2* was deleted, showing that acquisition of *URA3* expression is of epigenetic origin (Figure 1C and Table S2). While the fluorescence levels of both tdTomato fused proteins were close (Figure 1A), the frequency of *URA3* expression was closer to that of the control strain for the N-terminal fusion, suggesting that it had better protein functionality than the C-terminal fusion (Figure 1C and Table S2). We therefore performed the sorting experiments with the N-terminal fused protein.

**Figure 2:**
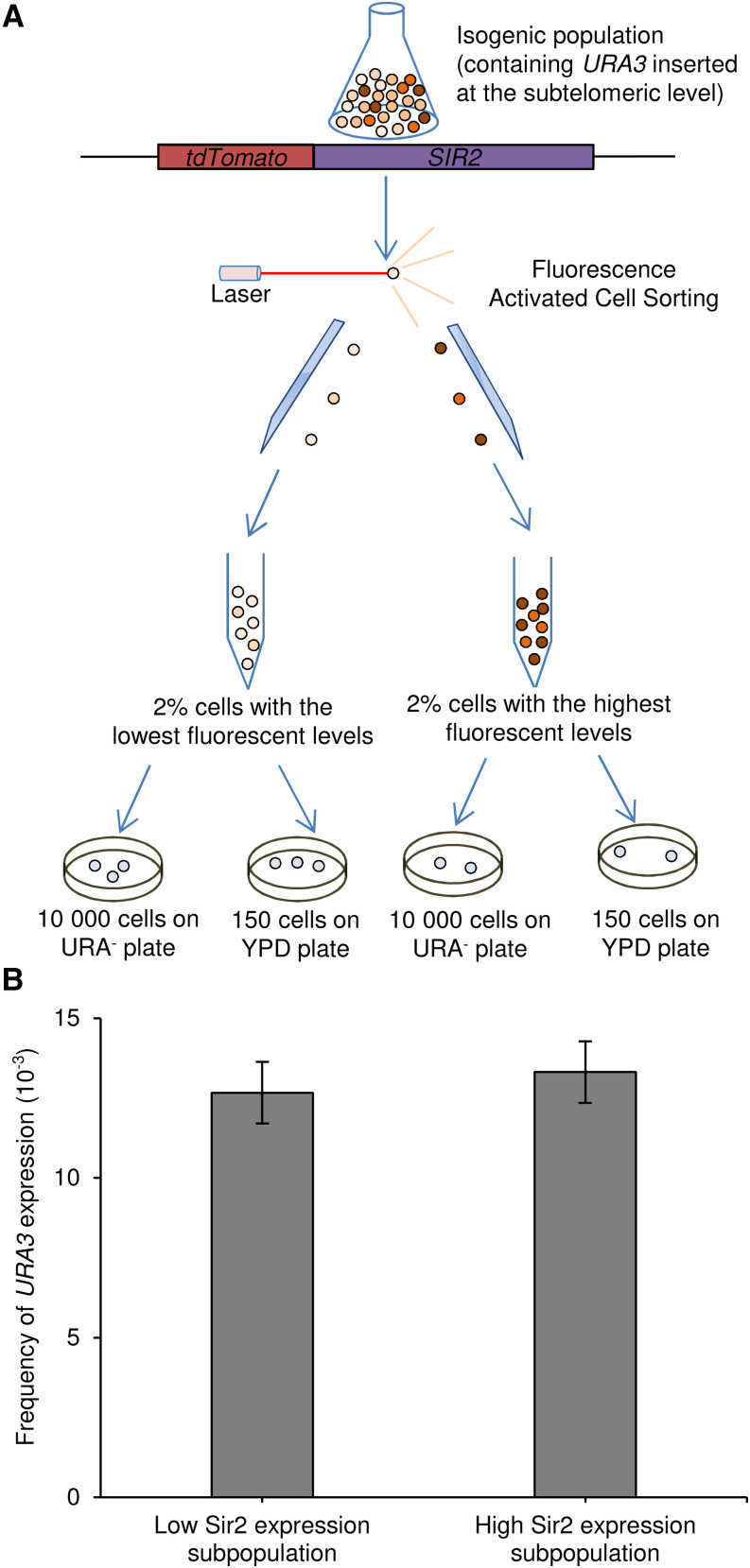
Noise in the expression of *SIR2* does not generate cell-to-cell heterogeneity in the frequency of *URA3* silencing., A) The fluorescent marker tdTomato fused N-terminally to *sir2* allows cells to be sorted by extreme expression levels (the highest 2% and the lowest 2%). A total of 10^4^ cells were sorted for each subpopulation and spread on SCD-Ura plates. Viability was evaluated in parallel on YPD plates, allowing the respective frequencies of *URA3* expression in these two subpopulations to be calculated., B) Frequency of *URA3* expression in the subpopulations with the highest (2%) and lowest (2%) cellular concentrations of tdTomato-Sir2. The results shown are the means and standard deviations of three independent experiments.

Among the cells with heterogeneous expression levels of *SIR2* at the single-cell level, we isolated the subpopulations (2%) with the highest and lowest fluorescence intensities (Figure 2A). The frequency of *URA3* expression was then evaluated using the *URA3* telomeric reporter by measuring the frequency of cells growing on SCD-ura plates normalized by the viability (Figure 2A). No difference between the Sir2-low and Sir2-high subpopulations was observed: the frequency of *URA3* expression was in both cases 1.4.10^−2^ (Figure 2B and Table S2), similar to one of the whole population with tdTomato-Sir2 (Figure 1C). We confirmed the epigenetic nature of *URA3* expression by re-spreading some of these clones on 5-FOA plates, where many cells were able to grow because of repressed *URA3* transcription (Figure S2). We also evaluated the time-dependence of the Sir2 level by looking at the dynamics of recovery of the expression profile after cell sorting for the low- and high-expressing cells. When grown in non-selective media, the initial distribution was not restored after 6 h (Figure S3 and Table S2) but was restored after 24 h (Figure S4) in both cases. During the initial phase of recovery, the low-expressors switched back to the initial distribution more rapidly than the high-expressors did, but still remained above the initial distribution after 6 h. Moreover, since the high-expressors remained far below the initial distribution after 6 h, the gap between high- and low-expressors was still large. Sir2 levels were thus relatively stable, at least in the Sir2-high subpopulation, and did not rapidly equilibrate in either subpopulation during the first 6 h of the initial experimental period. The initial expression profile was then recovered 24 h after sorting. However, considering that starving cells that are ura- in the first hours do not grow while URA+ cells start growing immediately, this difference of a few generations between initially ura- and URA+ cells should be reflected in the colony size after 3 days, the larger ones corresponding to initially URA+ cells. We therefore only scored large colonies. Switches that occurred after the first few hours were thus excluded (if new URA+ cells arose in the 6-24hr window as silencing reverts, they should have a difference of at least 3 to 12 generations compared to already URA+ cells). Nevertheless, it cannot be ruled out that the absence of a significant difference in silencing was at least partly due to the recovery of expression equilibrium during growth on SCD-ura plates. Finally, as the colony phenotypes attributed to reversion were not systematically verified on all colonies, it is not excluded that some of the small colonies can be attributed to slow growth of cells expressing *URA3* at low frequency in the strain lacking Ppr1 without silencing reversion, but it should not change the interpretation of our results.

**Figure 3:**
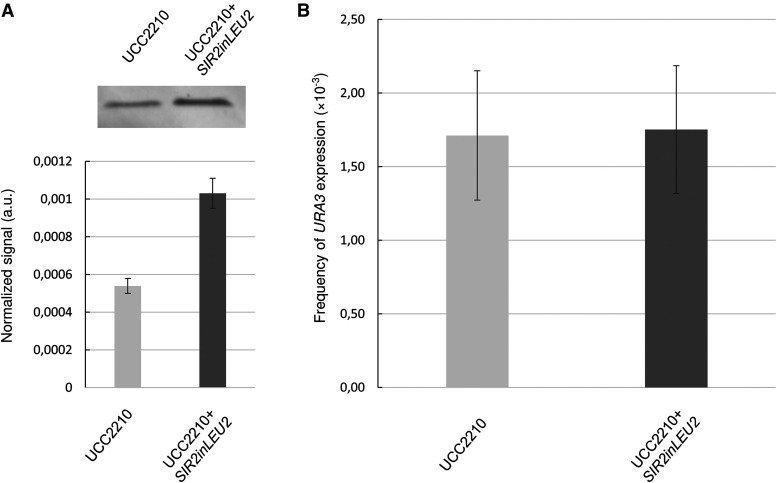
Doubling the mean expression level of Sir2 does not impact the frequency of *URA3* silencing., A) Western blot analysis and quantification of the level of Sir2 expression in the control strain (UCC2210) and the strain with an additional copy of *SIR2* inserted in the *LEU2* locus (UCC2210+*SIR2**in**LEU2*) using stain-free imaging technology and the software Image Lab (Bio-Rad). The results shown are the means and mean deviations of two independent experiments (raw data of Sir2 levels and total protein levels assessment together with an example of stain free staining are provided in Table S2)., B) Frequency of *URA3* expression in the control strain UCC2210 and the strain with an additional copy of *SIR2* in the *LEU2* locus (UCC2210+*SIR2**in**LEU2*). The results shown are the means and standard deviations of three independent experiments.

To test this hypothesis and confirm whether or not Sir2 was limiting for the silencing of telomeric *URA3* in our assays, we analyzed whether changes in Sir2 levels affected silencing without using cell sorting. To this end, we increased Sir2 levels by adding a copy of *SIR2* in the *LEU2* locus in the UCC2210 strain. The Western-blot data highlight the twofold increase in Sir2 levels compared to the original strain (Figure 3A and Table S2). Although this difference in mean expression is far from the ∼10-fold difference in tdTomato-Sir2 expression in low- and high-expressing subpopulations (Figure 1A), it appears that this increased mean Sir2 expression did not affect the silencing rate (Figure 3B and Table S2), showing that changes in mean Sir2 levels have no effect on the reporter gene in the absence of cell sorting.

### SIR2 expression noise can generate cell-to-cell heterogeneity in viability

**Figure 4:**
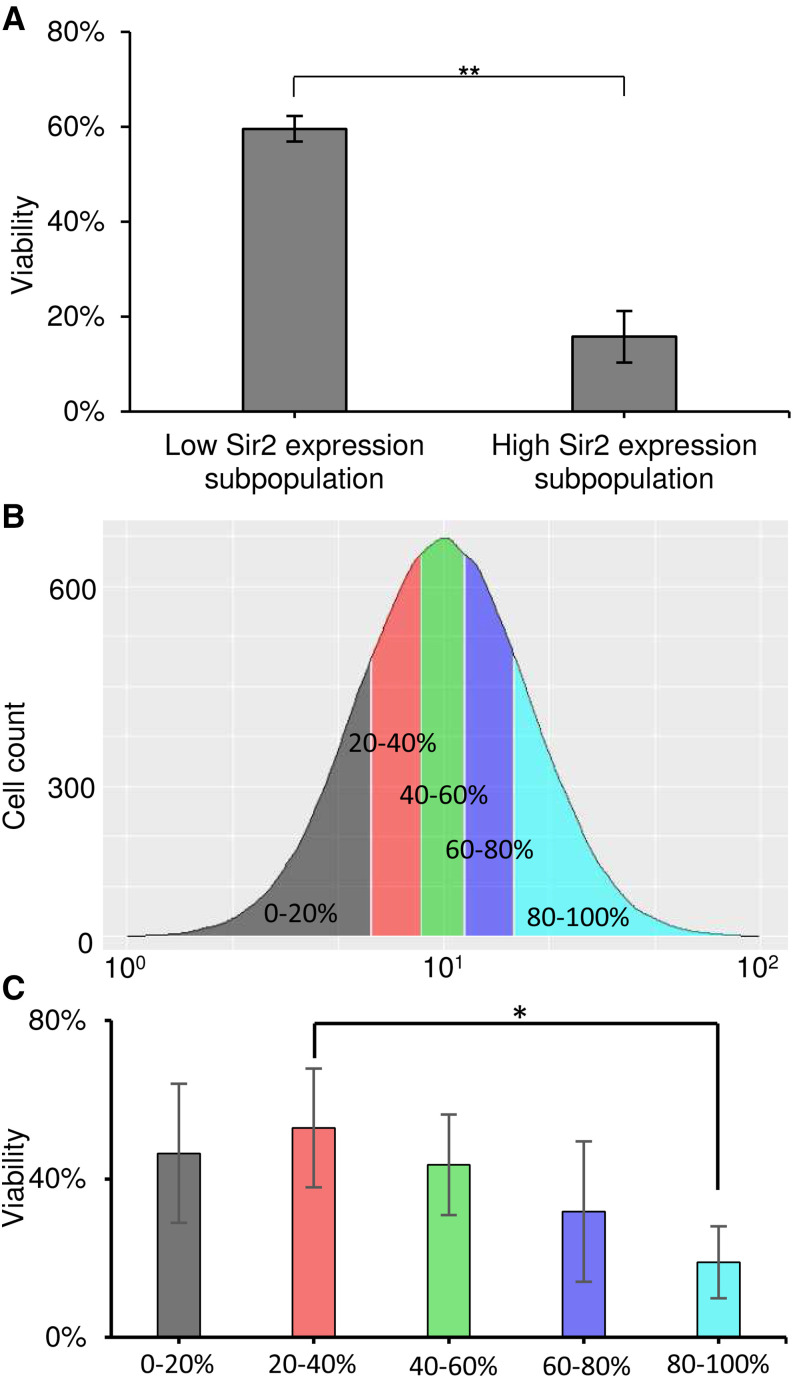
Noise in the expression of *SIR2* can generate cell-to-cell heterogeneity in viability., A) Viability in the different subpopulations used to measure frequencies of *URA3* expression in the UCC2210 strain (the 2% of cells with the lowest and highest Sir2 expression levels). The results shown are the means and standard deviations of three independent experiments. Statistically significant differences (*P* < 0.01, T tests) are indicated by double asterisks (**)., B) Five subpopulations, each representing 20% of the total population, homogenously distributed across the whole population and numbered 1 to 5 from the lowest to the highest expression levels, were sorted thanks to the fused protein tdTomato-Sir2. The same number of cells were sorted for each subpopulation and were spread on YPD plates (rich medium) to calculate viability. Three independent experiments were performed., C) Viability in these five subpopulations. The results shown are the means and standard deviations of three independent experiments. Statistically significant differences (*P* < 0.05, T tests) are indicated by single asterisks (*).

Surprisingly, when measuring viability to normalize the frequency of *URA3* expression, we observed a highly significant difference between the Sir2-low and Sir2-high subpopulations in our *URA3* telomeric reporter strain: while 6/10 Sir2-low cells were viable, this proportion was just 2/10 among Sir2-high cells (Figure 4A and Table S2) (*P* = 6.10^−3^). The two subpopulations behave very differently: their viability is strongly influenced by their cellular concentrations of tdTomato-Sir2. To make sure that this decrease in viability was not due to a toxic effect of tdTomato, we also sorted extreme subpopulations of a tdTomato-Tsl1 strain that contained tdTomato N-terminally fused to Tsl1 and that had the same fluorescence distribution as the strain containing tdTomato-Sir2 (Figure S5A). Contrary to the significant difference observed for Sir2 (*P* = 8.10^−3^), no difference in viability was observed between the extreme expression levels of tdTomato-Tsl1 (Figure S5B), suggesting that a toxic effect of tdTomato can be excluded. This strain was also used to verify that YPD and SCD media gave the same viability results, which is the case (Table S2).

Finally, to confirm the initial observation and to investigate whether viability scales linearly or non-linearly with Sir2 levels, we analyzed viability over the full range of Sir2 expression by dividing the whole population into five homogenously distributed subpopulations from the lowest (subpopulation 1) to the highest (subpopulation 5) expression levels (0–20%, 20–40%, etc.) (Figure 4B). Viability was negatively associated with Sir2 levels (Pearson r = -0.895) (Figure 4C and Table S2). While the difference was statistically significant only between subpopulations 2 and 5 (*P* = 0.02), viability scaled linearly with Sir2 levels: it was similar in the 0–20% and 20–40% subpopulations and then decreased linearly from the 20–40% subpopulation to the top 20%. These results show that Sir2 expression impacts viability not only at the highest expression levels but also at more moderate, non-extreme levels.

## Discussion

Gene expression is now recognized as a major source of phenotypic heterogeneity that can have profound consequences in various biological systems (Ackermann 2015). However, how epigenetics could be influenced by noise in the expression of the underlying genes has never been tested. Here, we tested the hypothesis that cell-to-cell variations in Sir2 levels could lead to differences in the frequency of epigenetic silencing in subtelomeric *URA3*. Our experiments allowed us to test the degree of cell-to-cell heterogeneity in epigenetic silencing due to stochastic *SIR2* expression variations in the vicinity of wild-type levels. The aim was to provide more information on the sensitivity of epigenetic silencing to stochastic variations in *SIR2* expression level. It appears that these fluctuations in *SIR2* expression levels are not sufficient to influence silenced states of subtelomeric *URA3*.

Elevated levels of Sir2 have been found to not increase the silencing of a telomeric *URA3* reporter gene (Renauld *et al.* 1993) or that of an *ADE2* reporter integrated at the *HMR* loci (Sussel *et al.* 1993), but have been shown to enhance repression of RNA pol II reporters in rDNA (Smith *et al.* 1998), with results indicating that rDNA silencing is highly sensitive to even small changes in Sir2 levels and that there are normally limited amounts of Sir2 available for rDNA silencing (Smith *et al.* 1998). Therefore, one possible explanation for the fact that Sir2 expression did not alter silencing in this study is that Sir2 was not limiting for the silencing of telomeric *URA3*. Indeed, we found that varying Sir2 levels did not change the silencing frequency of *URA3* at subtelomeres, in accordance with the literature. Nevertheless, other studies of strains carrying *ADE2* integrated next to the telomeric repeat of chromosome V-R have shown that Sir2 is normally limiting for maximal telomeric repression (Cockell *et al.* 2000), indicating a certain dependency on the reporter used. Thus, testing other genomic loci and other genes would also help to generalize our results and identify the specificity of each locus/gene pair. In any case, analyzing the global epigenetic landscape of the Sir2-low and Sir2-high subpopulations should provide information on the short-term sensitivity of the repression of chromatin to *SIR2* expression noise.

Moreover, the noise level of *SIR2* may itself be minimized as observed for essential genes and protein complex subunits (Fraser *et al.* 2004). There are no noise measurements from systematic studies available because its expression level is too low (Newman *et al.* 2006). Nevertheless, it encodes for a protein complex subunit and genes with similar functions such as *RPD3* whose noise has been measured indeed have low noise (Newman *et al.* 2006). This minimal noise would also minimize the epigenetic consequences of its expression fluctuations. Paradoxically, the targets of these chromatin remodelers are themselves expressed with high noise (Newman *et al.* 2006). Promoters with nucleosome binding sites generate higher cell-to-cell variability in gene expression (Sanchez and Golding 2013) while promoters with polynucleosome-disfavoring sequences have lower noise (Sharon *et al.* 2014). This intrinsic propensity of epigenetically regulated-genes to be noisy also suggests that noise in the expression of chromatin remodelers has to be minimized to limit downstream consequences.

Our results also show that *SIR2* expression noise can generate cell-to-cell heterogeneity in viability. The 2% of cells with the highest cellular concentrations of Sir2 were far less viable than the 2% with the lowest Sir2 levels in the telomeric *URA3* reporter strain, and this observation was confirmed by the general trend observed across the Sir2 expression range, with viability decreasing progressively with increasing Sir2 levels. These results, which indicate that high Sir2 subpopulations are less viable, seem contradictory to the observation that increased *SIR2* gene dosage extends replicative lifespan (Kaeberlein *et al.* 1999; Fahrenkrog 2015). However, it has also been reported that high levels of Sir2 expression can compromise viability (Holmes *et al.* 1997). An explanation that can be excluded is that the decreased viability is due to the toxicity of high levels of tdTomato because a strain containing another protein N-terminally fused to tdTomato (Tsl1) that is expressed with the same fluorescence distribution as tdTomato-Sir2 does not have such heterogeneity in viability between the least and most fluorescent cells (Figure S5).

The difference in viability is surprising given that we did not observe cell-to-cell heterogeneity in silencing, suggesting that the toxic effect may be independent of transcriptional silencing. One of the many mechanisms that have been proposed to explain Sir2-mediated extended lifespan (Kenyon 2010) is that it maintains gene silencing at telomeres during ageing (Dang *et al.* 2009). Nevertheless, high levels of Sir2 can be toxic to yeast and this effect seem rather due to decreased genome stability than to induced transcriptional silencing (Holmes *et al.* 1997). Note also that Sir2-induced lethality can be suppressed by H4 overexpression (Matecic *et al.* 2002). Thus, the viability issues caused by high Sir2 levels are almost certainly the result of a chromatin effect. In particular, if the cause is not promiscuous silencing of an essential gene, silencing of some other chromatin-based events, such as replication origins (Hoggard *et al.* 2018) may be involved and associated with genome instability.

While *Drosophila* experiments indicate that overexpression of *SIR2* promotes caspase-dependent apoptosis (Griswold *et al.* 2008), increased Sir2 activity prevents programmed cell death caused by osmotic stress in yeast (Vendrell *et al.* 2011), highlighting the fact that the underlying mechanisms by which *SIR2* expression or activation modulates survival remain largely unresolved. While the origins of the spontaneous cell death observed in microbial cultures even under optimal growth conditions are widely debated, multiple experimental data suggest that loss of genome integrity is a major source of apoptotic signals (Carmona-Gutierrez *et al.* 2010). Our work shows that noise in the expression of *SIR2* is a mechanism that can induce spontaneous apoptosis in a small extreme subpopulation, and previous results suggest that possible underlying mechanisms are decreased genome stability and/or enhanced apoptotic signals.

By examining epigenetic and phenotypic effects of cell-to-cell heterogeneity in *SIR2* expression, this work shows that these fluctuations are not sufficient to produce single-cell variability in the epigenetic silencing of subtelomeric *URA3* in *S. cerevisiae*, probably because Sir2 is not limiting for silencing subtelomeric *URA3*. Nevertheless, a strong effect on cell viability was observed with an almost linear relationship between decreased viability and increased Sir2 levels. The origins of this effect remain to be explored.

## References

[bib1] Ackermann M., 2015 A functional perspective on phenotypic heterogeneity in microorganisms. Nat. Rev. Microbiol. 13: 497–508. 10.1038/nrmicro3491 26145732

[bib2] Anderson M. Z., Gerstein A. C., Wigen L., Baller J. A., and Berman J., 2014 Silencing is noisy: population and cell level noise in telomere-adjacent genes is dependent on telomere position and sir2. PLoS Genet. 10: e1004436 10.1371/journal.pgen.1004436 25057900 PMC4109849

[bib3] Becskei A., Kaufmann B. B., and van Oudenaarden A., 2005 Contributions of low molecule number and chromosomal positioning to stochastic gene expression. Nat. Genet. 37: 937–944. 10.1038/ng1616 16086016

[bib4] Blake W. J., Balazsi G., Kohanski M. A., Isaacs F. J., Murphy K. F., 2006 Phenotypic consequences of promoter-mediated transcriptional noise. Mol. Cell 24: 853–865. 10.1016/j.molcel.2006.11.003 17189188

[bib5] Carmona-Gutierrez D., Eisenberg T., Buttner S., Meisinger C., Kroemer G., 2010 Apoptosis in yeast: triggers, pathways, subroutines. Cell Death Differ. 17: 763–773. 10.1038/cdd.2009.219 20075938

[bib6] Cockell M. M., Perrod S., and Gasser S. M., 2000 Analysis of Sir2p domains required for rDNA and telomeric silencing in Saccharomyces cerevisiae. Genetics 154: 1069–1083. 10757754 10.1093/genetics/154.3.1069 PMC1461001

[bib7] Dang W., Steffen K. K., Perry R., Dorsey J. A., Johnson F. B., 2009 Histone H4 lysine 16 acetylation regulates cellular lifespan. Nature 459: 802–807. 10.1038/nature08085 19516333 PMC2702157

[bib8] Dey S. S., Foley J. E., Limsirichai P., Schaffer D. V., and Arkin A. P., 2015 Orthogonal control of expression mean and variance by epigenetic features at different genomic loci. Mol. Syst. Biol. 11: 806 10.15252/msb.20145704 25943345 PMC4461400

[bib9] Fahrenkrog B., 2015 Histone modifications as regulators of life and death in Saccharomyces cerevisiae. Microb. Cell 3: 1–13. 10.15698/mic2016.01.472 28357312 PMC5354586

[bib10] Fraser H. B., Hirsh A. E., Giaever G., Kumm J., and Eisen M. B., 2004 Noise minimization in eukaryotic gene expression. PLoS Biol. 2: e137 10.1371/journal.pbio.0020137 15124029 PMC400249

[bib11] Giaever G., Flaherty P., Kumm J., Proctor M., Nislow C., 2004 Chemogenomic profiling: identifying the functional interactions of small molecules in yeast. Proc. Natl. Acad. Sci. USA 101: 793–798. 10.1073/pnas.0307490100 14718668 PMC321760

[bib12] Greenstein R. A., Jones S. K., Spivey E. C., Rybarski J. R., Finkelstein I. J., 2018 Noncoding RNA-nucleated heterochromatin spreading is intrinsically labile and requires accessory elements for epigenetic stability. eLife 7: e32948 10.7554/eLife.32948 30020075 PMC6070336

[bib13] Griswold A. J., Chang K. T., Runko A. P., Knight M. A., and Min K. T., 2008 Sir2 mediates apoptosis through JNK-dependent pathways in Drosophila. Proc. Natl. Acad. Sci. USA 105: 8673–8678. 10.1073/pnas.0803837105 18562277 PMC2438412

[bib14] Grunstein M., and Gasser S. M., 2013 Epigenetics in Saccharomyces cerevisiae. Cold Spring Harb. Perspect. Biol. 5: a017491 10.1101/cshperspect.a017491 23818500 PMC3685889

[bib15] Hoggard T. A., Chang F., Perry K. R., Subramanian S., Kenworthy J., 2018 Yeast heterochromatin regulators Sir2 and Sir3 act directly at euchromatic DNA replication origins. PLoS Genet. 14: e1007418 10.1371/journal.pgen.1007418 29795547 PMC5991416

[bib16] Holmes S. G., Rose A. B., Steuerle K., Saez E., Sayegh S., 1997 Hyperactivation of the silencing proteins, Sir2p and Sir3p, causes chromosome loss. Genetics 145: 605–614. 9055071 10.1093/genetics/145.3.605 PMC1207846

[bib17] Hornung G., Bar-Ziv R., Rosin D., Tokuriki N., Tawfik D. S., 2012 Noise-mean relationship in mutated promoters. Genome Res. 22: 2409–2417. 10.1101/gr.139378.112 22820945 PMC3514670

[bib18] Kaeberlein M., McVey M., and Guarente L., 1999 The SIR2/3/4 complex and SIR2 alone promote longevity in Saccharomyces cerevisiae by two different mechanisms. Genes Dev. 13: 2570–2580. 10.1101/gad.13.19.2570 10521401 PMC317077

[bib19] Kenyon C. J., 2010 The genetics of ageing. Nature 464: 504–512. 10.1038/nature08980 20336132

[bib20] Liu J., Francois J. M., and Capp J. P., 2019 Gene Expression Noise Produces Cell-to-Cell Heterogeneity in Eukaryotic Homologous Recombination Rate. Front. Genet. 10: 475 10.3389/fgene.2019.00475 31164905 PMC6536703

[bib21] Liu J., Martin-Yken H., Bigey F., Dequin S., Francois J. M., 2015 Natural yeast promoter variants reveal epistasis in the generation of transcriptional-mediated noise and its potential benefit in stressful conditions. Genome Biol. Evol. 7: 969–984. 10.1093/gbe/evv047 25762217 PMC4419794

[bib22] Lum P. Y., Armour C. D., Stepaniants S. B., Cavet G., Wolf M. K., 2004 Discovering modes of action for therapeutic compounds using a genome-wide screen of yeast heterozygotes. Cell 116: 121–137. 10.1016/S0092-8674(03)01035-3 14718172

[bib23] Mano Y., Kobayashi T. J., Nakayama J., Uchida H., and Oki M., 2013 Single cell visualization of yeast gene expression shows correlation of epigenetic switching between multiple heterochromatic regions through multiple generations. PLoS Biol. 11: e1001601 10.1371/journal.pbio.1001601 23843746 PMC3699475

[bib24] Matecic M., Stuart S., and Holmes S. G., 2002 SIR2-induced inviability is suppressed by histone H4 overexpression. Genetics 162: 973–976. 12399404 10.1093/genetics/162.2.973 PMC1462276

[bib25] Newcomb B., and Bedalov A., 2009 Identification of inhibitors of chromatin modifying enzymes using the yeast phenotypic screens. Methods Mol. Biol. 548: 145–160. 10.1007/978-1-59745-540-4_8 19521823 PMC2925398

[bib26] Newman J. R., Ghaemmaghami S., Ihmels J., Breslow D. K., Noble M., 2006 Single-cell proteomic analysis of S. cerevisiae reveals the architecture of biological noise. Nature 441: 840–846. 10.1038/nature04785 16699522

[bib27] Obersriebnig M. J., Pallesen E. M., Sneppen K., Trusina A., and Thon G., 2016 Nucleation and spreading of a heterochromatic domain in fission yeast. Nat. Commun. 7: 11518 10.1038/ncomms11518 27167753 PMC4865850

[bib28] Raj A., and van Oudenaarden A., 2008 Nature, nurture, or chance: stochastic gene expression and its consequences. Cell 135: 216–226. 10.1016/j.cell.2008.09.050 18957198 PMC3118044

[bib29] Rawal Y., Chereji R. V., Qiu H., Ananthakrishnan S., Govind C. K., 2018 SWI/SNF and RSC cooperate to reposition and evict promoter nucleosomes at highly expressed genes in yeast. Genes Dev. 32: 695–710. 10.1101/gad.312850.118 29785963 PMC6004078

[bib30] Renauld H., Aparicio O. M., Zierath P. D., Billington B. L., Chhablani S. K., 1993 Silent domains are assembled continuously from the telomere and are defined by promoter distance and strength, and by SIR3 dosage. Genes Dev. 7: 1133–1145. 10.1101/gad.7.7a.1133 8319906

[bib31] Sanchez A., Choubey S., and Kondev J., 2013 Regulation of noise in gene expression. Annu. Rev. Biophys. 42: 469–491. 10.1146/annurev-biophys-083012-130401 23527780

[bib32] Sanchez A., and Golding I., 2013 Genetic determinants and cellular constraints in noisy gene expression. Science 342: 1188–1193. 10.1126/science.1242975 24311680 PMC4045091

[bib33] Sharon E., van Dijk D., Kalma Y., Keren L., Manor O., 2014 Probing the effect of promoters on noise in gene expression using thousands of designed sequences. Genome Res. 24: 1698–1706. 10.1101/gr.168773.113 25030889 PMC4199362

[bib34] Simon J. A., and Bedalov A., 2004 Yeast as a model system for anticancer drug discovery. Nat. Rev. Cancer 4: 481–492. 10.1038/nrc1372 15170450

[bib35] Small E. C., Xi L., Wang J. P., Widom J., and Licht J. D., 2014 Single-cell nucleosome mapping reveals the molecular basis of gene expression heterogeneity. Proc. Natl. Acad. Sci. USA 111: E2462–E2471. 10.1073/pnas.1400517111 24889621 PMC4066511

[bib36] Smith J. S., Brachmann C. B., Pillus L., and Boeke J. D., 1998 Distribution of a limited Sir2 protein pool regulates the strength of yeast rDNA silencing and is modulated by Sir4p. Genetics 149: 1205–1219. 9649515 10.1093/genetics/149.3.1205 PMC1460222

[bib37] Sussel L., Vannier D., and Shore D., 1993 Epigenetic switching of transcriptional states: cis- and trans-acting factors affecting establishment of silencing at the HMR locus in Saccharomyces cerevisiae. Mol. Cell. Biol. 13: 3919–3928. 10.1128/MCB.13.7.3919 8321199 PMC359929

[bib38] To T. L., and Maheshri N., 2010 Noise can induce bimodality in positive transcriptional feedback loops without bistability. Science 327: 1142–1145. 10.1126/science.1178962 20185727

[bib39] Uphoff S., Lord N. D., Okumus B., Potvin-Trottier L., Sherratt D. J., 2016 Stochastic activation of a DNA damage response causes cell-to-cell mutation rate variation. Science 351: 1094–1097. 10.1126/science.aac9786 26941321 PMC4827329

[bib40] Vendrell A., Martinez-Pastor M., Gonzalez-Novo A., Pascual-Ahuir A., Sinclair D. A., 2011 Sir2 histone deacetylase prevents programmed cell death caused by sustained activation of the Hog1 stress-activated protein kinase. EMBO Rep. 12: 1062–1068. 10.1038/embor.2011.154 21836634 PMC3185340

[bib41] Viñuelas J., Kaneko G., Coulon A., Vallin E., Morin V., 2013 Quantifying the contribution of chromatin dynamics to stochastic gene expression reveals long, locus-dependent periods between transcriptional bursts. BMC Biol. 11: 15 10.1186/1741-7007-11-15 23442824 PMC3635915

[bib42] Weinberger L., Voichek Y., Tirosh I., Hornung G., Amit I., 2012 Expression noise and acetylation profiles distinguish HDAC functions. Mol. Cell 47: 193–202. 10.1016/j.molcel.2012.05.008 22683268 PMC3408861

[bib43] Xu E. Y., Zawadzki K. A., and Broach J. R., 2006 Single-cell observations reveal intermediate transcriptional silencing states. Mol. Cell 23: 219–229. 10.1016/j.molcel.2006.05.035 16857588

[bib44] Zaugg J. B., and Luscombe N. M., 2012 A genomic model of condition-specific nucleosome behavior explains transcriptional activity in yeast. Genome Res. 22: 84–94. 10.1101/gr.124099.111 21930892 PMC3246209

[bib45] Zhang Z., Qian W., and Zhang J., 2009 Positive selection for elevated gene expression noise in yeast. Mol. Syst. Biol. 5: 299 10.1038/msb.2009.58 19690568 PMC2736655

[bib46] Zoller B., Nicolas D., Molina N., and Naef F., 2015 Structure of silent transcription intervals and noise characteristics of mammalian genes. Mol. Syst. Biol. 11: 823 10.15252/msb.20156257 26215071 PMC4547851

